# Sacubitril/Valsartan Improves Left Atrial and Left Atrial Appendage Function in Patients With Atrial Fibrillation and in Pressure Overload-Induced Mice

**DOI:** 10.3389/fphar.2019.01285

**Published:** 2019-10-29

**Authors:** Ya Suo, Meng Yuan, Hongmin Li, Yue Zhang, Ying Li, Huaying Fu, Fei Han, Changhui Ma, Yuanyuan Wang, Qiankun Bao, Guangping Li

**Affiliations:** Tianjin Key Laboratory of Ionic-Molecular Function of Cardiovascular Disease, Department of Cardiology, Tianjin Institute of Cardiology, the Second Hospital of Tianjin Medical University, Tianjin, China

**Keywords:** sacubitril/valsartan, atrial fibrillation, left atrial function, left atrial appendage thrombus, atrial fibrosis

## Abstract

LCZ696 (sacubitril/valsartan) is an angiotensin receptor-neprilysin inhibitor and has shown beneficial effects in patients with heart failure. However, whether LCZ696 protects against left atrial (LA) and LA appendage (LAA) dysfunction is still unclear. The present study aimed to assess the efficacy of LCZ696 for improving the function of LA and LAA. We performed both a retrospective study comparing LCZ696 with angiotensin receptor blockers (ARBs) to assess the efficacy of LCZ696 in patients with atrial fibrillation and an animal study in a mouse model with pressure overload. LA peak systolic strain, LAA emptying flow velocity, and LAA ejection fraction (LAAEF) were significantly increased in patients with LCZ696 as compared with ARBs (p = 0.024, p = 0.036, p = 0.026, respectively). Users of LCZ696 had a lower incidence of spontaneous echocardiography contrast (p = 0.040). Next, patients were divided into two groups (LAAEF ≤ 20% and > 20%). Administration of LCZ696 in patients with LAAEF > 20% was more frequent than LAAEF ≤ 20% (p = 0.032). Even after controlling for LAA dysfunction-related risk factors (age, atrial fibrillation type, old myocardial infarction, hypertension, congestive heart failure, and prior stroke or transient ischemic attack), use of LCZ696 remained significantly associated with reduced probability of LAAEF ≤ 20% [odds ratio = 0.011; 95% confidence interval (0.000–0.533), p = 0.023]. To further confirmed effect of LCZ696 in LA function, we constructed a post-transverse aortic constriction model in mice. Mice with LCZ696 treatment showed lower LA dimension and higher left ventricular ejection fraction and LAA emptying flow velocity as compared with mice with vehicle or valsartan treatment. Meanwhile, as compared with vehicle or valsartan, LCZ696 significantly decreased LA fibrosis in mice. In summary, we provide evidence that LCZ696 may be more effective in improving LA and LAA function than ARBs in both humans and mice, which suggests that LCZ696 might be evaluated as a direct therapeutic for atrial remodeling and AF.

## Introduction

Patients with cardiogenic embolic stroke have a high prevalence of non-valvular atrial fibrillation (AF), which is associated with a five-fold increased risk of stroke and a two-fold increased risk of both dementia and mortality (Fuster et al., 2006). In AF patients, impaired atrial emptying might cause atrial blood stasis and enlargement of left atrial (LA), which could further offer a suitable terrain for thrombus formation. The left atrial appendage (LAA) is an important attachment and reservoir of LA, and its effective contraction can prevent blood stasis. AF confers an increased risk of stroke owing to the formation of atrial thrombus, usually in the LAA (Takada et al., 2001). Therefore, it is necessary to preserve the mechanical function of LAA to prevent LAA thrombosis and cardiogenic stroke in patients with AF (Perez et al., 1997; Verhorst et al., 1997). Several parameters measured by transesophageal echocardiography (TEE) can reflect LAA dysfunction (Zabalgoitia et al., 1998), which can result in LAA thrombus (LAAT) formation. Spontaneous echocardiographic contrast (SEC, caused by blood stasis or low-velocity blood flow), reduced LAA emptying flow velocity (LAAeV), and LAA ejection fraction (LAAEF) can be used as effective markers for stratifying thromboembolic risk in patients with AF (Kamp et al., 1999).

Activation of the renin-angiotensin-aldosterone system (RAAS) in atrial local tissue leads to structural and electrophysiological remodeling of the atrium, which further increases the susceptibility of atrial arrhythmia and promotes the occurrence of AF (Healey et al., 2005). LA and LAA function decline with mechanical remodeling of the left atrium, and thrombus is more likely to form within the LAA (Sparks et al., 1999). With atrial fibrosis, conduction abnormalities result in increased AF vulnerability (Jalife, 2014; Jalife and Kaur, 2015; Miragoli and Glukhov, 2015; Nattel, 2017). Recent studies have implicated that inhibition of RAAS activation can moderate atrial remodeling, thereby improving the occurrence and development of AF (Jibrini et al., 2008). In previous work, we demonstrated that administration of RAAS inhibitors in patients with AF might reduce the risk of LAAT by moderating atrial remodelling (Suo et al., 2018). In addition, natriuretic peptides (NPs) are critical regulators of cardiac structure and electrophysiology, modulating ion channel function in the heart, including the atrium (Moghtadaei et al., 2016). NPs also have anti-fibrotic effects (Rose and Giles, 2008), so they may play a protective role against AF development.

LCZ696 (sacubitril/valsartan) is a representative drug targeting both the RAAS and NP systems, which blocks the angiotensin receptor (AT1), and prevents the degradation of NPs *via* inhibiting neprilysin (Voors et al., 2013). In clinical studies, LCZ696 could improve New York Heart Association classification and cardio-renal function in patients with different types of heart failure, including heart failure with reduced left ventricular ejection fraction (HFrEF) and heart failure with preserved left ventricular ejection fraction (HFpEF) (Solomon et al., 2012; Balmforth et al., 2019). In the PARAMOUNT study, patients with LCZ696 showed a significant decrease in LA diameter than patients with valsartan (Solomon et al., 2012). However, whether the administration of LCZ696 could protect against LA and LAA dysfunction has not been elucidated.

Therefore, we undertook this work including both a retrospective study comparing LCZ696 with AT1 blockers (ARBs) to assess the efficacy of LCZ696 in patients with AF and an animal study in a mouse model with pressure overload. LCZ696 was more effective than ARBs in preserving LA and LAA function. Even when controlling for risk factors associated with LAA dysfunction, use of LCZ696 remained significantly associated with reduced risk of LAAEF ≤ 20%. In addition, we verified that LCZ696 improved LA and LAA function and decreased atrial fibrosis in mice with pressure overload. Thus, LCZ696 might have potential therapeutic value in preventing the incidence of cardiogenic embolic stroke in patients with AF.

## Materials and Methods

### Study Population

We conducted a retrospective study of all consecutive adult AF patients admitted to the Second Hospital of Tianjin Medical University from October 1, 2017, to March 31, 2019, and underwent both TEE and transthoracic echocardiography (TTE) on the same day. The total number of all patients was 468, and 81 patients were eventually included in the analysis on the basis of exclusion criteria (see below). AF and its different types were defined according to ESC guidelines for the management of AF (Kirchhof et al., 2016) The researchers were unaware of the results and recorded the required data, including demographic details, LAAT-related risk factors, and LCZ696 or ARB use. Patients who used LCZ696 or ARBs for more than 12 weeks were assigned to the LCZ696 group and ARB group, respectively. LCZ696 (cat. no. H20170362) and valsartan (cat. no. H20040217) were purchased from Novartis Pharma Schweiz AG. The CHA_2_DS_2_–VASc [congestive heart failure, hypertension, age ≥75 years (doubled), diabetes mellitus, prior stroke or transient ischemic attack or thromboembolism (doubled), vascular disease, age 65 to 74 years, sex category] point score system was used for stratifying ischemic stroke risk among patients with non-valvular AF. Other clinical data including vascular disease (peripheral arterial disease, carotid disease, venous thromboembolic disease, or renal artery stenosis), hyperlipidemia (hypercholesterolemia, hypertriglyceridemia, mixed hyperlipidemia, and low-/high-density lipoproteinemia), smoking history (active smoking history accumulated over 6 months), drinking history (alcohol average >120 g/week), and anti-platelet drug use (aspirin and/or clopidogrel for > 12 weeks) were collected.

**Figure 1:**
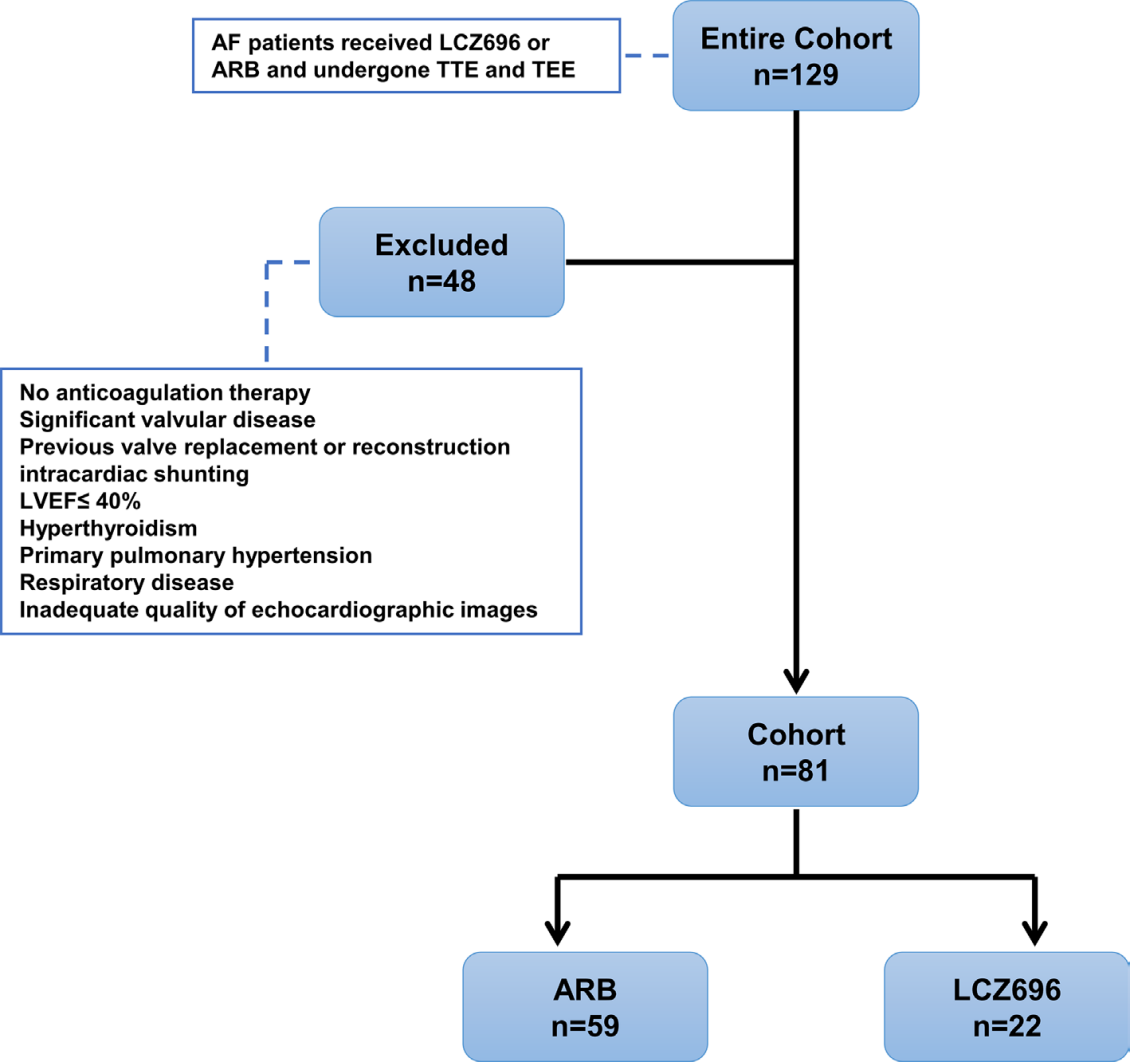
Study design displaying inclusion/exclusion and patient group assignments. AF, atrial fibrillation; ARB, angiotensin receptor blocker; TTE, transthoracic echocardiography; TEE, transesophageal echocardiography; LVEF, left ventricular ejection fraction.

Patients who did not receive anti-coagulant therapy were excluded from this study. Other exclusion criteria were acute myocardial infarction, significant valvular disease, congenital heart disease, left ventricular (LV) systolic dysfunction [LV ejection fraction (LVEF) ≤ 40%], severe respiratory disease, pulmonary hypertension, and inadequate quality of echocardiographic images. The dysfunction of LA and LAA was the outcome of this study. The Local Ethics Committee approved the study protocol, with all study subjects providing written informed consent (**Figure 1**).

### Echocardiography Measurements

Echocardiography examination involved using a commercial ultrasonography system (IE33, Philips Healthcare, Inc.). TTE involved using a 1–5 MHz phased S5-1 probe, and TEE a 2–7 MHz 3D matrix array X7-2t probe. All images were analyzed by using off-line post-processing with QLAB Software.

The following parameters were evaluated in standard views with standard techniques (Lang et al., 2005): LV end-diastolic diameter (LVDd), LVEF, LA dimension (LAD), maximal LA volume (LAVmax), and ratio of early transmittal flow velocity and early mitral annular velocity (E:e’ ratio). Spectral Doppler tissue imaging was used to measure tissue Doppler velocity; the septal annuli was selected in this study. LA was divided into 13 segments, and LA peak systolic strain during ventricular systole was calculated by the mean of all 13 segments.

**Figure 2:**
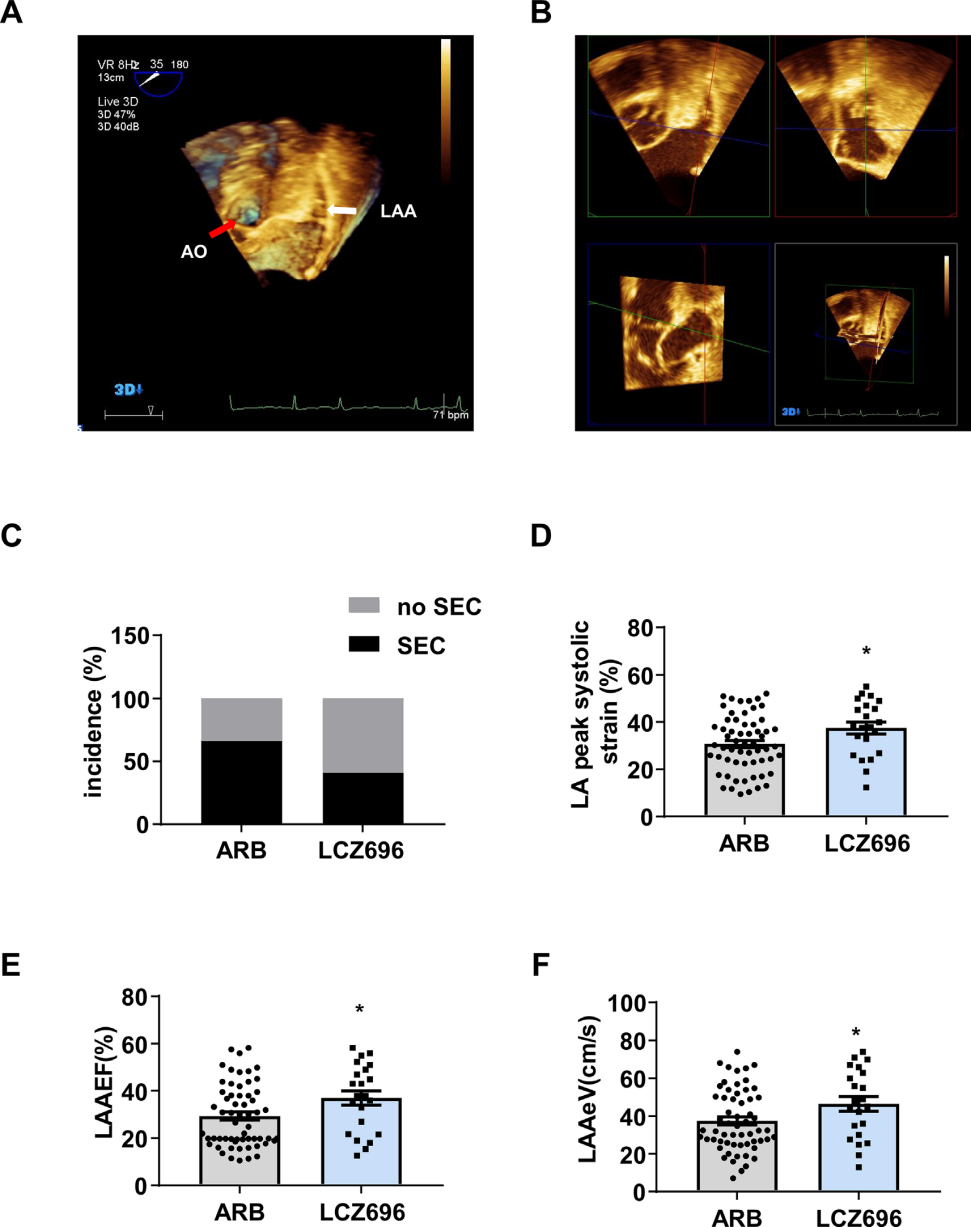
Sacubitril/valsartan is more effective than angiotensin receptor blockers for improving left atrial and left atrial appendage function in patients with atrial fibrillation.**(A)** 3D transesophageal echocardiography (3D-TEE) showing the left atrial appendage (LAA) from the basal short-axis view in the transverse scan. The white arrow indicates the LAA, and the red arrow indicates the aorta (AO). **(B)** QLAB 3DQ plug-in showing LAA. The LAA short axes were aligned by using QLab-3DQ software, allowing visualization of the LAA oriﬁce in the multiplanar reconstruction mode. **(C)** Incidence of spontaneous echocardiographic contrast (SEC), **(D)** LA peak systolic strain, **(E)** LAA ejection fraction (LAAEF), and **(F)** LAA emptying flow velocity (LAAeV). Data are mean ± SD, *P < 0.05, unpaired two-tail *t* test.

An echocardiographer reviewed all TEE images to determine whether LAAT, SEC, reduced LAAeV, and LAAEF were present. The echocardiographer was blinded to the clinical data and results of TTE. LAAT was defined as a uniformly and circumscribed echo-dense intra-cavitary mass in multiple imaging planes, distinct from the pectinate muscles, and LAA endocardium (Aschenberg et al., 1986). SEC was defined as dynamic “smoke-like” echoes with the characteristic swirling motion during the entire cardiac cycle (Fatkin et al., 1994). The LAAeV was obtained at a depth of 1/3 from the LAA orifice with pulsed Doppler. Then, a 3D-TEE study was performed to visualize various LAA structures. LAAEF was calculated by using the QLAB 3DQ plug-in. We measured LAA volume from the basal short-axis view in the transverse scan. LAAEF was calculated as [(maximum volume - minimum volume)/maximum volume × 100%] (**Figures 2A, B**). The researcher carefully measured the parameters and calculated the mean values from five cardiac cycles.

### Animal Study

Animal procedures were approved and conducted in accordance with the Experimental Animal Administration Committee of Tianjin Medical University. Male C57BL/6J mice at 8–10 weeks of age were randomly assigned to transverse aortic constriction (TAC) surgery or sham surgery. All mice were housed in a controlled environment (20 ± 2°C, 12-hr/12-hr light/dark cycle). TAC surgery was performed as described (Wang et al., 2016). Briefly, after mice were anesthetized, the transverse aorta was ligated with a 27-G needle. Mice in the sham surgery group underwent similar surgical procedures without ligation of the transverse aorta. At 8 weeks after surgery, TAC mice were randomized to treatment with LCZ696 (60 mg/kg body weight perorally, n = 6), valsartan (48 mg/kg body weight perorally, n = 6), or no treatment (n = 6) for another 4 weeks. Mice were given valsartan or LCZ696 dissolved in corn oil or only corn oil every day by oral gavage.

### Echocardiography Measurements of Mice

**Figure 3:**
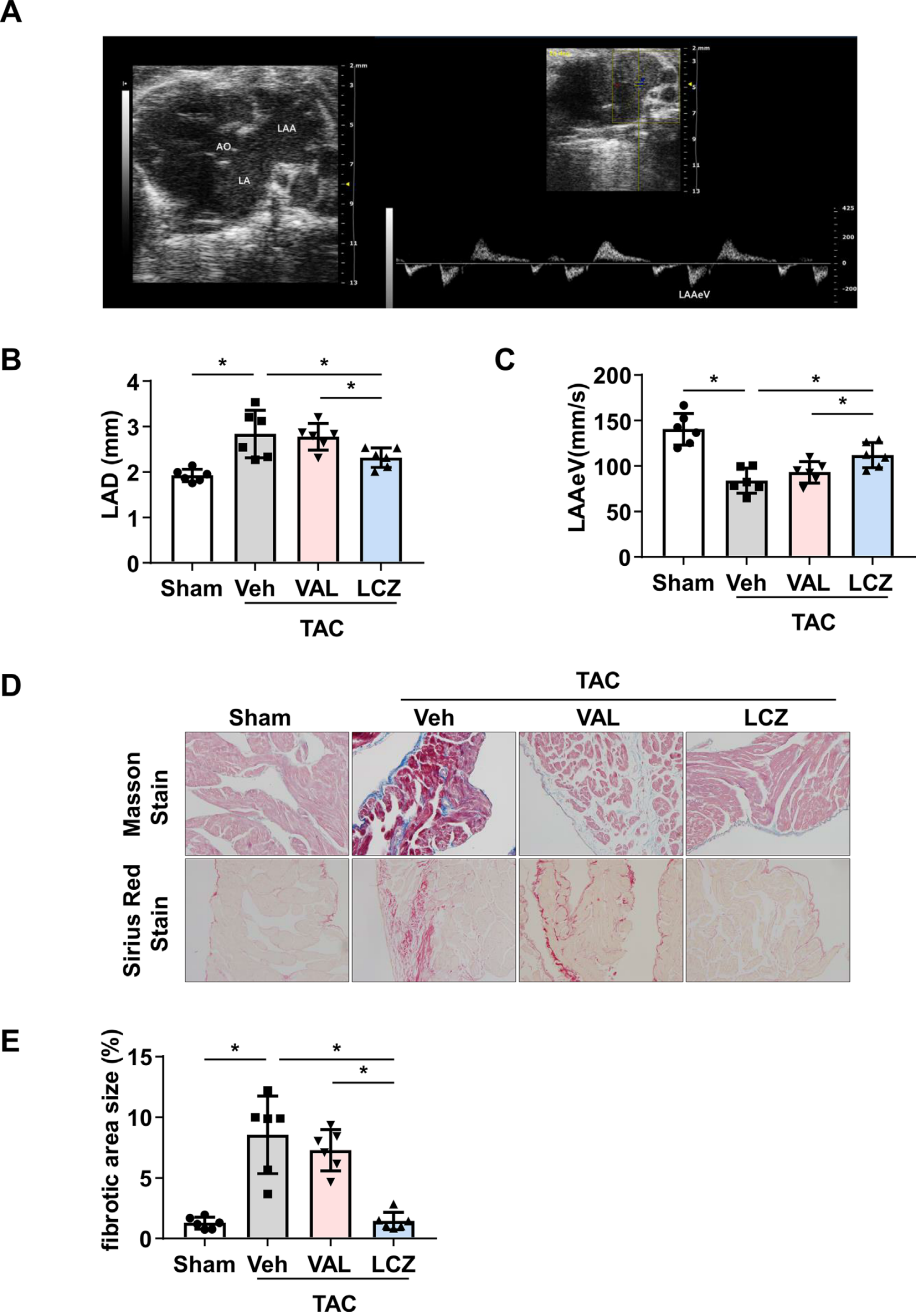
Sacubitril/valsartan is more effective than valsartan for protecting left atrial (LA) and left atrial appendage (LAA) function and decreasing LA fibrosis in mice after pressure overload.**(A)** 2D echocardiography showing LA, LAA, aorta (AO), and LAA emptying flow velocity (LAAeV) from the basal short-axis view. **(B, C)** Left atrial dimension (LAD) and LAAeV quantified by echocardiography. **(D)** Representative images of Masson staining and Sirius Red staining of LA. Scale bar, 100 μm. **(E)** Quantification of the fibrotic area in **(D)**. TAC, transverse aortic constriction; Veh, vehicle; VAL, valsartan; LCZ, LCZ696. Data are mean ± SEM, n = 6 mice per group, *P < 0.05, one-way ANOVA.

TTE was performed on all mice at baseline (8 weeks post-TAC surgery) and 4 weeks post-randomization by using a Vevo 2100 System with an MS400 Linear Array Transducer (VisualSonics, ON, Canada) as described (Gao et al., 2011) After mice were weighed and anesthetized by inhalation of 1.5% isoflurane, they were placed on a heating mat to maintain normothermia (35°C). LAD, LV interventricular septum thickness diameter (IVSTd), LV posterior wall thickness diameter (LVPWd), LVDd, and LVEF were measured. LAAeV was obtained on the parasternal short-axis view (**Figure 3A**). After final echocardiography examination, mice were immediately sacrificed for heart tissues.

### Histology of Heart Tissues

Heart tissues were fixed in 10% neutral-buffered formalin for 24 h at room temperature and embedded in paraffin, then sectioned at 5 µm for staining. Collagen deposition was stained with Masson’s trichrome stain (Sigma-Aldrich, MO) and Sirius Red stain (Solarbio Life Sciences, Beijing) according to the manufacturer’s instructions. Images of the sections were captured under an Olympus Inverted Microscope (IX53, Tokyo) and fibrotic areas were semi-quantitatively determined by using ImageJ v1.52.

### Statistical Analyses

All statistical analyses were performed with SPSS v23.0 (SPSS, Chicago, IL, USA). Results are presented as mean ± standard deviation (SD) or mean±SEM for continuous variables and percentage of the total number of patients for categorical variables. Student *t*-test was used to compare continuous variables. Chi-square and the Fisher exact test were used for nominal variables. Univariate analysis was performed with the chi-square test. Variables significant on univariate logistic regression analysis (*p* < 0.05) were entered into the multiple regression analysis. Logistic regression analysis (with the enter method) was performed to identify independent predictors of depressed LAAEF. The risk was expressed as odd ratios (OR) with 95% confidence intervals (CIs). Statistical analysis involved use of GraphPad Prism 7 (Version 7.04), by one-way ANOVA. The criterion for statistical significance was *P* < 0.05.

## Results

### Sacubitril/Valsartan Is More Effective Than Angiotensin Receptor Blockers for Improving Left Atrial and Left Atrial Appendage Function in Patients With Atrial Fibrillation

Initially, we identified 129 patients with AF who used LCZ696 or ARBs and received TTE and TEE during hospitalization from 2017 to 2019; 81 were included in the analysis: 22 (27.2%) received LCZ696 and 59 (72.8%) received ARBs (**Figure 1**). The mean age of 81 patients was 62.76 ± 8.19 years; 55.6% were male (**Table 1**). The baseline clinical characteristics were comparable between the two groups (**Table 1**). The two groups did not differ in LVDd, LVEF, LAD, LAVmax, or E:e’ ratio (**Table 2**). However, users of LCZ696 had a lower incidence of SEC and higher LAAeV, LAAEF, and LA peak systolic strain as compared with users of ARBs (**Table 2**, **Figure 2**). LAAT occurred in 21% of 81 patients, including 22.0% users of ARBs and 18.2% users of LCZ696 (*p* = 0.705) (**Table 2**).

**Table 1:** Baseline characteristics of sacubitril/valsartan and angiotensin receptor blocker users.

Variables	LCZ696 n = 22	ARBs n = 59	*P* value
Age, years	60.454 ± 8.410	63.627 ± 8.014	0.135
Age ≥ 65 years, %	31.8%	47.5%	0.206
Male sex, %	63.6%	52.5%	0.371
BMI, kg/m^2^	25.865 ± 3.669	26.189 ± 3.785	0.731
AF type, %			0.933
Paroxysmal AF	68.2%	71.2%	
Persistent AF	22.7%	22.0%	
Long-standing persistent	9.1%	6.8%	
OMI, %	27.3%	16.9%	0.351
Vascular disease, %	4.5%	5.1%	1.000
CHD, %	90.9%	71.2%	0.062
Hyperlipidemia, %	68.2%	66.1%	0.860
Hypertension, %	59.1%	71.2%	0.300
Diabetes mellitus, %	36.4%	23.7%	0.255
Congestive heart failure, %	22.7%	13.6%	0.325
Prior stroke or TIA, %	27.3%	20.3%	0.554
CHA_2_DS_2_–VASc Score, mean±SD	2.454 ± 1.738	2.644 ± 1.471	0.625
Smoking history, %	45.5%	39.0%	0.598
Drinking history, %	27.3%	22.0%	0.621
Antiplatelet drugs, %	68.2%	55.9%	0.318

BMI, body mass index; OMI, old myocardial infarction; CHD, coronary heart diseases; TIA, transient ischemic attack; AF, atrial fibrillation; CHA_2_DS_2_–VASc Score, congestive heart failure, hypertension, age ≥75 years (doubled), diabetes mellitus, prior stroke or transient ischemic attack or thromboembolism (doubled), vascular disease, age 65 to 74 years, sex category.

**Table 2:** Echocardiographic parameters for sacubitril/valsartan and angiotensin receptor blocker users.

Variables	LCZ696 n = 22	ARB n = 59	*P* value
LAD, mm	38.814 ± 2.910	40.244 ± 3.960	0.127
LAVmax, ml	48.713 ± 11.652	52.630 ± 13.926	0.244
LA peak systolic strain, %	37.536 ± 11.679	30.727 ± 11.892	0.024
LVDd, mm	50.075 ± 5.460	49.468 ± 5.950	0.677
LVEF, %	53.163 ± 6.456	56.558 ± 7.095	0.053
E:e’ ratio	11.040 ± 4.404	12.420 ± 4.327	0.208
LAAeV, cm/s	46.459 ± 18.250	31.481 ± 16.363	0.036
LAAEF, %	37.050 ± 14.289	29.413 ± 13.215	0.026
SEC, %	40.9%	66.1%	0.040
LAAT, %	18.2%	22.0%	0.705

Data are mean ± SD unless indicated. LAD, left atrial dimension; LAVmax, maximal left atrial volume; LVDd, left ventricular end-diastolic diameter; LVEF, left ventricular ejection fraction; E,e’ ratio, the ratio of the early transmitral flow velocity and the early mitral annular velocity; LAAeV, left atrial appendage emptying flow velocity; LAAEF, left atrial appendage ejection fraction; SEC, spontaneous echocardiographic contrast; LAAT, left atrial appendage thrombus.

Next, the 81 patients were divided into two groups according to the cutoff for LAAEF defined as 20% (LAAEF ≤ 20% and > 20%) (Iwama et al., 2012). Demographic, echocardiographic parameters and clinical characteristics of the patients with LAAEF≤ 20% or > 20% are shown in **Table 3**. Patients with LAAEF > 20% were younger than those with LAAEF ≤ 20% (61.4 ± 8.6 *vs.* 65.2 ± 6.9 years) (*p* < 0.05) (**Table 3**). CHA_2_DS_2_-VASc score was significantly higher for patients with LAAEF ≤ 20% than > 20% (*p*< 0.05). As compared with patients with LAAEF > 20%, those with LAAEF ≤ 20% had a higher prevalence of long-standing persistent AF (continuous AF >12 months duration), persistent AF (continuous AF sustained < 7 days), old myocardial infarction (OMI), prior stroke or transient ischemic attack, congestive heart failure, and hypertension. The proportion of LCZ696 users was higher for patients with LAAEF > 20% than ≤ 20% (35.3 *vs.* 13.3%) (*p* < 0.05). LAAeV, LVEF, and LA peak systolic strain were significantly lower for patients with LAAEF ≤ 20% than > 20%. Patients with LAAEF ≤ 20% had significantly higher E:e’ ratio, LAD, LAVmax, LVDd, and incidence of SEC and LAAT than those with LAAEF > 20% (*p* < 0.01) (**Table 3**).

**Table 3:** Clinical and echocardiography parameters for patients with left atrial appendage ejection fraction ≤ 20% or LAAEF > 20%.

Variables	LAAEF ≤ 20% n = 30	LAAEF > 20% n = 51	*P* value
Age, years	65.17 ± 6.85	61.35 ± 8.64	0.042
Age≥65 years, %	63.3%	31.4%	0.005
Male sex, %	65.5%	56.3%	0.238
BMI, kg/m^2^	26.10 ± 3.5	26.1 ± 3.90	0.996
AF type, %			0.000
Paroxysmal AF	40.0%	88.2%	
Persistent AF	43.3%	9.8%	
Long-standing persistent AF	16.7%	2.0%	
OMI, %	33.3%	11.8%	0.019
Vascular disease, %	6.7%	3.9%	0.624
CHD, %	80.0%	74.5%	0.573
Hyperlipidemia, %	70.0%	64.7%	0.625
Hypertension, %	93.3%	52.9%	0.000
Diabetes mellitus, %	20.0%	31.4%	0.266
Congestive heart failure, %	30.0%	7.8%	0.013
Prior stroke or TIA, %	42.3%	9.8%	0.000
CHA_2_DS_2_–VASc score	3.57 ± 1.406	2.02 ± 1.32	0.000
Smoking history, %	36.7%	43.1%	0.567
Drinking history, %	20.0%	25.5%	0.573
Antiplatelet drugs, %	66.7%	54.9%	0.298
LCZ696, %	13.3%	35.3%	0.032
LAD, mm	42.55 ± 3.20	38.27 ± 3.08	0.000
LAVmax, ml	59.3 ± 13.8	47.02 ± 10.94	0.000
LA peak systolic strain, %	22.27 ± 8.79	38.64 ± 9.5	0.000
LVDd, mm	51.62 ± 6.67	48.47 ± 4.91	0.017
LVEF, %	52.5 ± 7.5	57.48 ± 6.13	0.002
E:e’ ratio	14.01 ± 4.24	10.89 ± 4.04	0.001
LAAeV, cm/s	24.79 ± 8.83	48.82 ± 14.6	0.000
LAAEF, %	17.18 ± 3.09	39.90 ± 10.32	0.000
SEC, %	100%	35.3%	0.000
LAAT, %	50.0%	3.9%	0.000

Data are mean ± SD unless indicated.

LAAT, left atrial appendage thrombus; BMI, body mass index; OMI, old myocardial infarction; CHD, coronary heart diseases; TIA, transient ischemic attack; AF, atrial fibrillation; LAD, left atrial dimension; LAVmax, maximal left atrial volume; LVDd, left ventricular end-diastolic diameter; LVEF, left ventricular ejection fraction; E,e’ ratio, the ratio of the early transmitral flow velocity and the early mitral annular velocity; LAAeV, left atrial appendage emptying flow velocity; LAAEF, left atrial appendage ejection fraction; SEC, spontaneous echocardiographic contrast; LAAT, left atrial appendage thrombus.

Logistic regression analysis was performed to identify independent clinical predictors of LAAEF ≤ 20% (**Table 4**). Univariate analysis demonstrated prior stroke or transient ischemic attack, congestive heart failure, categories of AF type, OMI, hypertension, and age as significantly positively associated with LAAEF ≤ 20%, except for use of LCZ696, which was significantly negatively associated with LAAEF ≤ 20%. However, only prior stroke or transient ischemic attack, AF type, hypertension, age ≥ 65 years, and use of LCZ696 were associated with LAAEF ≤ 20% after multivariable adjustments (**Table 4**). Hypertension was associated with a higher risk of LAAEF≤ 20% [OR = 9.797; 95% CI (1.202–79.883); *p* = 0.033]. Congestive heart failure and OMI were not significantly correlated with LAA dysfunction. After controlling for factors related to LAAEF, the use of LCZ696 remained significantly associated with reduced probability of LAAEF ≤ 20% [OR = 0.011; 95% CI (0.000–0.533), *p* = 0.023).

**Table 4:** Factors associated with left atrial appendage emptying flow velocity ≤ 20% on multiple regression analyses.

Variables	Odds ratio	95% CI	*P* value
Age ≥ 65 years	7.675	1.183–49.805	0.033
AF type			0.007
OMI	5.465	0.461–64.757	0.178
Hypertension	9.797	1.202–79.883	0.033
CHF	3.095	0.127–75.434	0.488
Prior stroke or TIA	10.474	1.305–76.018	0.027
LCZ696	0.011	0.000–0.533	0.023

LAAEF, left atrial appendage ejection fraction; OMI, old myocardial infarction; CHF, congestive heart failure; TIA, transient ischemic attack; CI, confidence interval.

### Sacubitril/Valsartan Was More Effective Than Valsartan in Protecting Left Atrial and Left Atrial Appendage Function and Decreasing LA Fibrosis in Mice With Pressure Overload

To detect the efficiency of LCZ696 in protecting LA and LAA function, we used the TAC mouse model, a pressure overload-induced LA and LAA dysfunction model. TAC surgery increased LAD, LVSTd, LVPWd, LVDd, and LVDs as compared with sham mice (**Table 5**). In addition, LAAeV and LVEF were significantly decreased after 8 weeks of TAC surgery.

**Table 5:** Baseline echocardiography parameters for mice under transverse aortic constriction surgery for 8 weeks.

Variables	Sham	TAC		
		Vehicle	Valsartan	LCZ696
LVSTd, mm	0.611 ± 0.038	0.827 ± 0.064*	0.813 ± 0.040*	0.801 ± 0.051^*^
LVPWd, mm	0.624 ± 0.036	0.818 ± 0.048*	0.856 ± 0.049*	0.800 ± 0.048*
LVDd, mm	3.330 ± 0.187	3.714 ± 0.195*	3.673 ± 0.362*	3.724 ± 0.312*
LVDs, mm	2.156 ± 0.211	2.907 ± 0.120*	2.882 ± 0.311*	2.895 ± 0.196*
LVEF, %	65.739 ± 6.776	44.477 ± 5.286*	44.339 ± 5.850*	45.315 ± 4.888*
LAD, mm	1.900 ± 0.105	2.236 ± 0.345*	2.222 ± 0.241*	2.221 ± 0.266*
LAAeV, mm/s	155.158 ± 17.184	125.858 ± 9.976*	122.349 ± 15.125*	127.932 ± 24.030*

Data are mean ± SEM.

LVSTd, left ventricular interventricular septum thickness diameter; LVPWd, left ventricular posterior wall thickness diameter; LVDd, left ventricular end-diastolic diameter; LVDs, left ventricular end-systolic diameter; LVEF, left ventricular ejection fraction; LAD, left atrial dimension; LAAeV, left atrial appendage emptying flow velocity. *P< 0.05 versus sham.

Next, mice were treated with LCZ696, valsartan, or vehicle for another 4 weeks. LAD was decreased and LAAeV increased in mice with LCZ696 as compared with vehicle and valsartan (**Table 6** and **Figures 3B, C**), which suggests that LCZ696 is more effective than valsartan in protecting LA and LAA function. Consistent with a previous report in rabbits (Torrado et al., 2018), in mice, LVEF was significantly improved with LCZ696 as compared with valsartan (**Table 6**). Moreover, Masson and Sirius red stain demonstrated that LCZ696 reduced fibrosis in the LAA induced by pressure overload as compared with valsartan (**Figures 3D, E**). Together, our data indicate that LCZ696 might be more effective in attenuating LA and LAA dysfunction induced by pressure overload than valsartan in mice.

**Table 6:** Echocardiography analysis of pressure overload-induced mice after 4-week treatment.

Variables	Sham	TAC		
		Vehicle	Valsartan	LCZ696
IVSTd, mm	0.632 ± 0.025^§^	1.005 ± 0.106*^§^	0.911 ± 0.078*^#^	0.865 ± 0.055*^#^
LVPWd, mm	0.630 ± 0.062^#§^	1.021 ± 0.101*^§^	0.961 ± 0.057*	0.887 ± 0.036*^#^
LVDd, mm	3.462 ± 0.223^#§^	4.177 ± 0.213*^§^	3.981 ± 0.362*	3.821 ± 0.222*^#^
LVDs, mm	2.360 ± 0.187^#§^	3.356 ± 0.198*^§^	3.197 ± 0.282*^§^	2.922 ± 0.224*^#^
LVEF, %	60.526 ± 7.265^#§^	40.508 ± 6.427*^§^	40.866 ± 3.219*^§^	47.759 ± 3.904*^#^
LAD, mm	1.927 ± 0.137^#^	2.842 ± 0.524*^§^	2.781 ± 0.293*^§^	2.318 ± 0.213^#^
LAAeV, mm/s	140.700 ± 17.257^#§^	84.079 ± 13.869*^§^	93.286 ± 11.785*^§^	112.170 ± 13.688*^#^

Data are mean ± SEM.

IVSTd, left ventricular interventricular septum thickness diameter; LVPWd, left ventricular posterior wall thickness diameter; LVDd, left ventricular end-diastolic diameter; LVDs, left ventricular end-systolic diameter; LVEF, left ventricular ejection fraction; LAD, left atrial dimension; LAA eV, LAA emptying flow velocity. *P< 0.05 versus Sham. ^#^P< 0.05 versus TAC + vehicle. §P< 0.05 versus LCZ696.

## Discussion

In the present study, we found that the use of LCZ696 was associated with improved LA and LAA function in patients with AF evaluated by echocardiography. In addition, LCZ696 was more effective than valsartan at moderating atrial fibrosis and protecting LA and LAA function in mice with pressure overload. Therefore, our study demonstrated a critical role for LCZ696 in protecting against LA/LAA structural remodeling and dysfunction.

Structural and electrophysiological remodeling of the atrial myocardium increases the susceptibility to arrhythmia and AF development (Burstein and Nattel, 2008). Fibrosis is an important pathophysiological basis of structural and electrophysiological remodelling (Burstein and Nattel, 2008). Atrial fibrosis leads to mechanical dysfunction of the atria and blood stasis in the atria, which leads to thrombosis in the LAA (Kamp et al., 1999; Sparks et al., 1999). The activation of RAAS results in adverse hemodynamic effects, hypertrophy, and especially the stimulation of fibrosis (Burstein and Nattel, 2008). Therefore, RAAS inhibitors containing angiotensin-converting enzyme inhibitors or ARBs are involved in the upstream treatment of AF. Inhibiting RAAS can reduce atrial fibrosis and dilated atrial volume, further delaying AF development (Nakashima and Kumagai, 2007). Our previous retrospective study demonstrated that the incidence of LAAT was reduced and echocardiography indicators related to left atrial remodeling were improved in patients with angiotensin-converting enzyme inhibitor/ARB treatment (Suo et al., 2018).

LCZ696 contains valsartan and the neprilysin inhibitor pro-drug sacubitril (AHU-377, which can be converted by enzymatic cleavage into the active neprilysin inhibiting metabolite LBQ657) in one compound (Gu et al., 2010). Neprilysin degrades biologically active NPs, including atrial NP, B-type natriuretic peptide, and C-type natriuretic peptide, which are critical regulators of atrial electrophysiology and structure (Moghtadaei et al., 2016). In contrast to the cardiovascular effects of RAAS, the NP system of hormones protects cardiac structure and function (Nathisuwan, 2002). Atrial deficiency and low levels of atrial natriuretic peptide were found associated with atrial fibrosis and AF recurrence (van den Berg et al., 2004). The effects of the NP system on cardiac remodeling have been implicated as a possible mechanism (van den Berg et al., 2004; Nishikimi et al., 2006). Therefore, NPs might play a protective role in AF development. Angiotensin II, endothelin 1, and transforming growth factor-β1 (TGF-β1) promote hypertrophic and fibrotic actions in cardiomyocytes and fibroblasts, whereas NPs *in vivo* and *in vitro* were shown to antagonize angiotensin II, endothelin 1 and TGF-β1 (Tamura et al., 2000; Nishikimi et al., 2006). However, neprilysin can degrade angiotensin II as well. Thus, a neprilysin inhibitor cannot be used to treat AF in monotherapy because activation of angiotensin II signaling pathways limits its beneficial effect in a feedback increase of NP levels (Richards et al., 1993). Simultaneous addition of ARBs and a neprilysin inhibitor to culture media of rat fibroblasts and cardiomyocytes was more effective than ARBs alone in inhibiting biochemical indicators of cardiac fibrosis and hypertrophy (von Lueder et al., 2013). Therefore, inhibition of both RAAS and neprilysin might have greater potential clinical benefit for neprilysin inhibitors in AF treatment.

LA strain assessed by 2D speckle tracking was inversely correlated with the burden of LA fibrosis analyzed by MRI (Kuppahally et al., 2010). Moreover, LA peak systolic strain can independently predict LA reverse mechanical and structural remodelling (Machino-Ohtsuka et al., 2013). These results suggest that LA peak systolic strain is related to LA function, according to the principle of strain, and useful for evaluating LA reservoir function (Kokubu et al., 2007). LAA acts as an important attachment and reservoir of LA, and its effective contraction can prevent blood stasis and LAAT formation (Tabata et al., 1998; Al-Saady et al., 1999). Moreover, LAA plays an important pathophysiological function when left atrial volume and pressure overload (Nakajima et al., 2010). LAAEF was an independent determinant of LAAT according to previous multivariate logistic regression analysis. And a LAAEF of 20% was the optimal cutoff value for predicting LAAT (Iwama et al., 2012). Compared with 2D imaging, 3D TEE imaging shows the clearer stereoscopic anatomical structure of LAA and more effective evaluation of the LAA function. Lower LAEF and LAAeV in AF patients were associated with increased risk of SEC and LAAT formation (Petersen et al., 2015).

In this study, patients using LCZ696 showed a lower incidence of SEC and greater LA peak systolic strain and LAAeV as compared with ARB users. LAAEF in patients receiving LCZ696 was significantly increased. Multiple regression analysis indicated LCZ696 independently associated with increased LAAEF. These results suggest that LCZ696 can improve LA and LAA function. However, we found no significant differences in incidence of LAAT, LAD, and LAVmax between the two groups. Since atrial dysfunction may occur before atrial structural changes in AF patients, a longer follow-up time is needed to observe atrial structural changes.

We also found hypertension as an independent predictor of LAAEF ≤ 20% [OR = 9.797; 95% CI (1.202–79.883); *p* = 0.033]. Regression models showed no interaction between hypertension and LCZ696 use. This result was consistent to the results of PARAMOUNT study, which demonstrated that the benefit of LCZ696 for reducing LAD and N-terminal pro-brain natriuretic peptide level were independent of the blood pressure-lowering effect. Although LCZ696 showed a better anti-hypertensive effect than valsartan, the two drugs were similar in tolerability (Ruilope et al., 2010; Solomon et al., 2012).

The study contains two limitations. First, we performed a hospital-based retrospective study and could not assess the dosage-related effects. Thus, larger, prospective studies are needed to ascertain the benefit of LCZ696 in AF. Second, the underlying molecular mechanism of the beneficial effect of LCZ696 is still unknown. In summary, this study showed that LCZ696 might be associated with improved LA and LAA function and had potential therapeutic value in preventing the incidence of cardiogenic embolic stroke in patients with AF.

## Data Availability Statement

The raw data supporting the conclusions of this manuscript will be made available by the authors, without undue reservation, to any qualified researcher.

## Ethics Statement

The studies involving human participants were reviewed and approved by the Experimental Animal Administration Committee of Tianjin Medical University. The patients/participants provided their written informed consent to participate in this study. The animal study was reviewed and approved by the Experimental Animal Administration Committee of Tianjin Medical University.

## Author Contributions

QB and GL contributed conception and design of the study. CM and YW organized the database. YS and HL performed the statistical analysis. YS and MY wrote the first draft of the manuscript. YZ, YL, HF, and FH wrote sections of the manuscript. All authors contributed to manuscript revision, read and approved the submitted version.

## Funding

This work was supported by grants from the National Natural Science Foundation of China (81800251, 81570304, 81800297).

## Conflict of Interest

The authors declare that the research was conducted in the absence of any commercial or financial relationships that could be construed as a potential conflict of interest.

## Abbreviations

AF, atrial fibrillation; ARB, angiotensin receptor blocker; BMI, body mass index; CHD, coronary heart diseases; E:e’ ratio, The ratio of the early transmitral flow velocity and the early mitral annular velocity; LA, left atrial; LAA, left atrial appendage; LAAEF, left atrial appendage ejection fraction; LAAeV, left atrial appendage emptying flow velocity; LAAT, left atrial appendage thrombus; LAD, left atrial dimension; LAVmax, maximal left atrial volume; LVDd, left ventricular end-diastolic diameter; LVDs, left ventricular end-systolic diameter; LVEF, left ventricular ejection fraction; LVPWd, left ventricular posterior wall thickness diameter; LVSTd, left ventricular interventricular septum thickness diameter; NPs, natriuretic peptides; OMI, old myocardial infarction; RAAS, renin-angiotensin-aldosterone system; SEC, spontaneous echocardiographic contrast; TAC, transverse aortic constriction; TEE, transesophageal echocardiography; TGF-β1, transforming growth factor-β1; TIA, transient ischemic attack; TTE, transthoracic echocardiography.
